# Patterns of Self-Medication Behavior for Oral Health Problems Among Adults Living in Riyadh, Saudi Arabia

**DOI:** 10.3390/pharmacy6010015

**Published:** 2018-02-01

**Authors:** Arwa Aldeeri, Haya Alzaid, Renad Alshunaiber, Shahad Meaigel, Naila A. Shaheen, Abdallah Adlan

**Affiliations:** 1 College of Dentistry, Riyadh Elm University, Riyadh 12734, Saudi Arabia; HayaAlzaid@gmail.com; 2 College of Dentistry, King Saud Bin-Abdulaziz University for Health Sciences, Riyadh 14811, Saudi Arabia; RenadAbdullaziz@gmail.com (R.A.); ShahadMeaigel@gmail.com (S.M.); 3 Department of Biostatistics and Bioinformatics, King Abdullah International Medical Research Center, Riyadh 14611, Saudi Arabia; AshrafNa@ngha.med.sa; 4 King Saud Bin-Abdulaziz University for Health Sciences, Riyadh 14811, Saudi Arabia; AdlanA@ngha.med.sa; 5 Department of Biomedical Ethics, King Abdullah International Medical Research Center, Riyadh 14611, Saudi Arabia

**Keywords:** self-medication, behavior, dental problems, oral health problems

## Abstract

Abstract: Self-medication is a widespread behavior worldwide. It is defined as the practice of self-diagnosis and drug prescription without proper professional consultation. **Aim:** To determine the prevalence and predictors of self-medication for oral health problems among adults living in Riyadh city. **Methods:** A cross-sectional study based on a structured close-ended questionnaire was distributed among adults visiting shopping malls in all different five regions of Riyadh. A two-stage sampling technique was used: cluster and simple random sampling. The questionnaire was composed of two main sections: demographic characteristics and questions assessing the behavior of self-medication. **Results:** The prevalence of self-medication was found to be 63.25%, with a higher prevalence among females than males. Gender and nationality were significantly associated with self-medication. Salt in hot water locally (52.57%) and acetaminophen (47.43%), a type of an analgesic, were, systemically, the most frequently used. Pharmacy shops were the main source of these medicaments (66.01%). Similarly, the advice for using them was mainly given by pharmacists (53.36%). Lack of time was claimed to be the main reason for practicing self-medication (54.55%) with abscess, toothache, and gingival bleeding being the main predictors. **Conclusions:** Self-medication was found to be a common practice among the population of Riyadh city.

## 1. Introduction

Self-medication is defined as the practice of self-diagnosis and drug prescription without seeking professional healthcare advice [1,2,3]. Worldwide, it is a common reported behavior [4,5]. Headache, cough, fever, and pain were the most common issues for practicing self-medication [6,7]. Additionally, oral health complaints were also a reason for pursuing this behavior such as toothache, gingival bleeding, halitosis, gingival swelling, tooth mobility and others [8,9]. Numerous studies reported that self-medication is a common behavior in both developed and developing courtiers [4,5,10]. For instance, In European countries, the prevalence of self-medication for general health issues is 68% [11], while it was found to be 94.5% in Saudi Arabia, 92% in Kuwait, 76% in Karachi, 55% in Egypt, and 44.8% in Bahrain [7,12,13,14]. Furthermore, in Cameroon a total of 67.8% were found to be self-medicating for oral health problems, a finding that supports similar reports in Indian and Nigerian populations, 100% and 80.6%, respectively [15,16]. In Saudi Arabia, 80% of the population were found to be self-medicating for dental pain of which antibiotics were the main medications used [9]. In contrast, a survey investigated a sample of Saudi papulation revealed a more positive attitude in regard to antibiotics of which 77.8% of the sample used them according to the doctor’s prescription only [17]. Several substances were used without professional consultation. These substances ranged from herbs and traditional recipes to pharmacological drugs. They include analgesics, the most commonly used medication, followed by native herbs, antibiotics, water with salts, oils, and others [8,18,19]. Approaching these substances was found to be through different, sources such as pharmacies, native healers, and herbal shops. Nevertheless, this behavior can result in deleterious events, such as drug interactions, toxicity, and bacterial resistance, all of which are caused primarily by poor diagnosis, inappropriate indication, and overdosing [20,21]. Moreover, sources of information and advice of the medications used for self-medication varied widely to include pharmacists, relatives, friends, media sites, traditional practitioners, and person’s own knowledge and experience. Shifting towards self-diagnosis and medication instead of seeking a proper professional healthcare is attributed to a lack of time, money, or healthcare accessibility, as well as religious or cultural beliefs, and receiving a previous treatment for a similar condition [8,15]. However, findings of the previous studies cannot be generalized as the samples were either dental patients or university students, which do not accurately represent the Saudi population. In addition, most of the reported studies were limited to investigate the practice of self-medication using antibiotics only [9,17]. The current study aims to assess the patterns of self-medication for the management of oral health problems among adults living in Riyadh, Saudi Arabia.

## 2. Methods

At first, an ethics statement was conducted in full accordance with the World Medical Association Declaration of Helsinki. It was independently reviewed and approved by the ethics committee at King Abdullah International Medical Research Center (KAIMRC), (IRB/RSS17/004/R). In August 2017, an observational cross-sectional study using self-administered, close-ended questionnaire adapted by Agbor et al. [8] was conducted. The questionnaire was composed of the demographic characteristics and items related to assessing the behavior of self-medication, including type of oral/dental problems they previously experienced, sources of medication recommendation, types of medication used, source and routes of medicament administration, duration of use, duration of symptoms relief, and reasons for not seeking proper dental consultation. The questionnaire was distributed in both Arabic and English versions to people visiting shopping malls. The shopping malls were selected from all regions of Riyadh city. The participants were selected at random during their visit to the shopping malls. Participants of any age, gender, or nationality were selected. No incentives were provided upon participation. In addition, participants who have never experienced oral health problems were excluded. 

The definition of self-medication was explained to the visitors as well as the aim of the current study. The visitors’ consent of participation was obtained verbally. Afterwards, they were handed iPads to sign the consent form before they filled out the questionnaire. Privacy and confidentiality were completely protected, no identifiers or information were collected, including participants names, IDs, and others. Gender, nationality, marital status, education, occupation, household income, region of living in Riyadh, and type of oral health problem were compared using a chi-square test. Logistic regression was used to identify the predictors of self-medication. The data was analyzed using SAS V9.3 (SAS Institute Inc., Cary, NC, USA). 

## 3. Results

A total of 400 participants were enrolled, of which 184 (46%) were males and 216 (54%) were females. The participants were classified into four age groups: 18–25 years, 134 (33.5%); 26–35 years, 155 (38.75%); 36–45 years, 66 (16.5%); and 45 years and older, 45 (11.25%). More than two thirds of the sample, 310 (77.5%), were Saudi. According to the marital status, 180 (45%) participants were single, while 220 (55%) were married. 

For the majority of the participants, 257 (64.25%), their educational level was above high school, while 143 (35.75%) were below high school. The classification of white collar jobs includes: teachers, sales representatives, doctors, attorneys, accountants, engineers, shopkeepers, and architects, of which 264 (66%) participants were included in this category. On the other hand, blue collar jobs include: policemen, painters, truck drivers, and heavy machine operators, of which it included 27 (6.75%) participants. Moreover, 109 (27.25%) participants were unemployed or retired. 

According to the household income, 46 (11.5%) participants their income was <3000 SR, 77 (19.25%) between 3000–4999 SR, 90 (22.5%) between 5000–8999 SR, 83 (20.75%) between 10,000–14,999 SR, and for 86 (21.5%) their income was more than 15,000 SR. According to the region of living in Riyadh city, 82 (20.5%) participants live in the east, 75 (18.75%) in the west, 95 (23.75%) in the north, 91 (22.75%) in the south, 47 (11.75%) in the middle, and 10 (2.5%) in rural regions. Upon their previous experience of oral problems, toothache and abscesses were experienced by 306 (76.5%) and 68 (17%) participants, respectively, *p*-value < 0.05 (Table 1). 

The overall prevalence of self-medication in the general population was found to be 63.25%. Across genders, females were found to be more self-medicating than males, 69.44% and 55.98%, respectively. Toothache was the main trigger for practicing self-medication, 201 (79.45%), followed by gingival bleeding, 64 (25.30%); dental abscess, 32 (12.65%); gingival swelling, 31 (12.25%); mouth ulcer, 14 (5.35%); bad breath, 13 (5.14%); and others, 9 (3.56%), including tooth mobility and caries.

**Figure 1:**
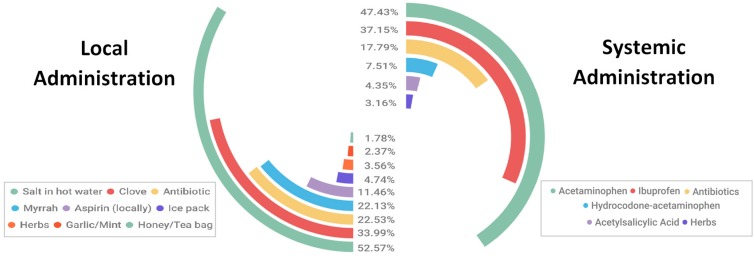
Types of used medications.

According to the route of administration, medications used without proper dental consultation were classified into locally- and systemically-administered medications. Salt in hot water was the main medication used locally (52.57%), followed by clove (33.99%) and topically applied antibiotics (22.53%), while acetaminophen was the main medication introduced systemically (47.43%), followed by Ibuprofen (37.15%) and antibiotics (17.79%) (Figure 1). The majority of the sample (70.75%) indicated the usage of the above-mentioned medications was for less than a week followed by one week (13.04%), two weeks (6.32%), 3–4 weeks (3.56%), 5–8 weeks (2.77%), and 3.65% of the participants used them for a duration of more than eight weeks. 

Symptom relief was experienced by 86.90% of the sample. However, 10.71% reported no effect, while the symptoms got worse with 2.38% of the participants. When the participants were asked about the next step in case their symptoms did not resolve or become better, 80.56% stated that they will visit a dentist, 9.52% will continue the same medication, 3.97% will visit a physician, and 5.56% will do nothing. 

**Figure 2:**
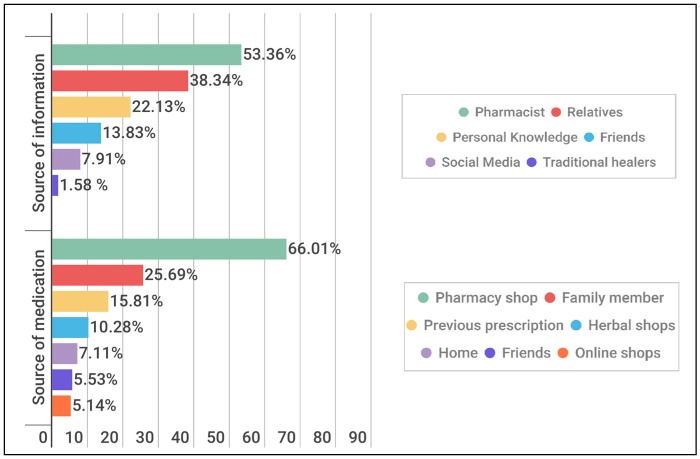
Sources of medications and recommendation.

**Figure 3:**
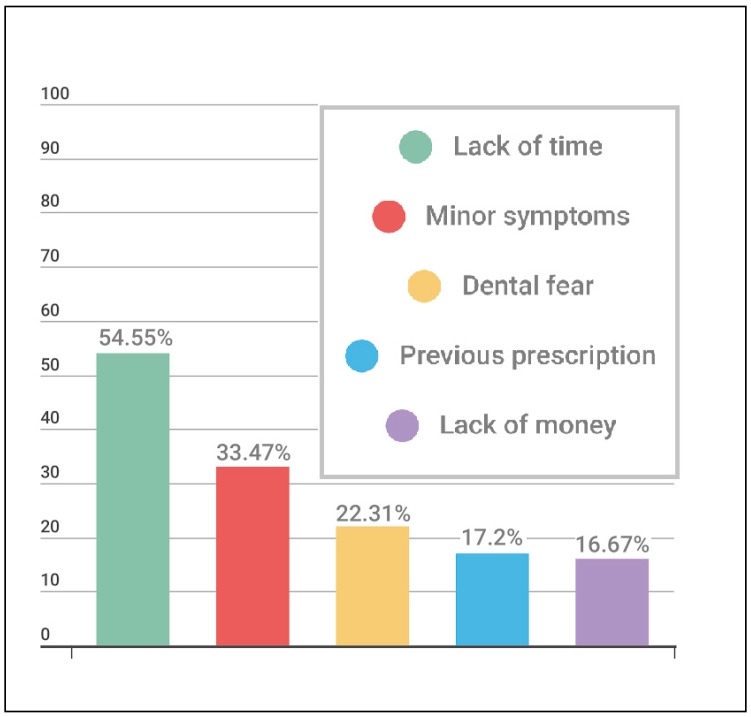
Reasons for practicing self-medication.

Pharmacists were the main source of recommending the usage of these medications (53.36%) with several sources being chosen of which pharmacy shops were the main source (66.01%) (Figure 2). Several reasons for practicing this behavior are shown in Figure 3 with lack of time being the main reason (54.55%). Many variables were assumed to be predictors for practicing self-medication of which toothache, abscess and gingival bleeding showed significant *p*-values < 0.05 (Table 2).

## 4. Discussion

The prevalence of self-medication to treat oral and dental problems in Riyadh city was found to be high (63.25%). This finding supports the global reported prevalence in the literature, 100% in India [15], 80.6% in Nigeria [16], 80% in Saudi Arabia [9], 67.8% in Cameroon [8], and 21.7% in Brazil [22]. Nevertheless, the prevalence of self-medication behavior for general health problems is significantly high. It was found to be 77.9% in the Greek population [23], 68% in France [11], 55.8% in Spain [24], and 23.6% in the United States [25]. To the best of our knowledge, this is the first study investigating the independent utilization of different types of medications for oral health problems in Riyadh, Saudi Arabia. In the current study, females were found to be significantly more self-medicating than males (*p* = 0.005). In contrast, similar studies found that gender was insignificantly associated with the practice of self-medication [8,16]. However, in another study among Saudi patients, self-medication using antibiotics only was significantly higher in males than females, 63.8% and 16.5%, respectively (*p* = 0.043) [9]. As for the triggers for practicing self-medication, in this study toothache was the main trigger (79.45%), which is in agreement with the findings among Indian (52.6%) and Cameroonian (54.7%) populations [8,15]. Moreover, another study reported a similar trigger for using antibiotics without proper dental consultation (86%) [9]. In agreement with the current study findings, analgesics were the main medications used among Indian (48%) and Cameroonian (40.9%) populations, followed by herbs, 29.7% and 26.8%, respectively. However, the usage of salt in hot water was limited among both populations to 8% and 3.3%, respectively [8,15]. In contrast, amoxicillin was the main medication used independently in Brazil (34.8%), followed by acetaminophen (32.6%) [22]. Pharmacy shops were found to be the main source for medications (66.01%). However, similar findings might indicate this issue to be experienced worldwide as reported in previous studies: Saudi Arabia (93.6%) [9], India (86%) [15], Cameroon (55.6%) [8], and Brazil (45.7%) [22]. The duration for practicing self-medication by the majority of the sample was less than a week (70.75%) which agrees with the finding in Indian population (60.6%) [15] and disagreement with Nigerian population, of which the majority practiced self-medication for 2–3 weeks (37.3%) [16]. In addition, most of the sample (86.90%) reported a symptoms relief which supports the finding in Cameroonian population (86.4%) [8]. However, this number has dropped to the half in Indian population (45.1%) [15]. Many reasons were claimed by the participants for practicing this behavior, of which lack of time was the main reason (54.55%) followed by their belief of the minority of the symptoms (33.47%) and dental fear (22.31%), with the latter being a serious issue receiving extensive investigations [26]. In case of symptom persistence, the majority of the participants will seek a dentist (80.56%), which is agreed upon by Indian population as well (84.6%) [15].

## 5. Conclusions

Self-medication was found to be a common practice among the population of Riyadh. This could be problematic because of possible complications and the delay of proper intervention. Educational campaigns to raise the awareness of potential hazards of self-medication and consequences of delaying the intervention among general population are needed. Further studies, including other populations of the Kingdom of Saudi Arabia, to detect other reasons for practicing this behavior and possible other predictors are recommended.

## Figures and Tables

**Table 1:** Demographic characteristics of the study cohort by status self-medication vs. no self-medication.

Variables	Self-Medication *n* (%) *n* = 253(63.25)	Non-Self Medication *n* (%) *n* = 147(36.75)	*p*-Value
Gender			0.005 *
Male	103 (40.71)	81 (55.10)	
Female	150 (59.29)	66 (44.90)	
Age			0.795
18–25	83 (32.81)	51 (34.69)	
26–35	101 (39.92)	54 (36.73)	
36–45	43 (17)	23 (15.65)	
46 and above	26 (10.28)	19 (12.93)	
Nationality			0.049 *
Saudi	204 (80.63)	106 (72.11)	
Non-Saudi	49 (19.37)	41 (27.89)	
Marital status			0.810
Single	115 (45.45)	65 (44.22)	
Married	138 (54.55)	82 (55.78)	
Educational Level			0.596
High school and below	88 (34.78)	55 (37.41)	
Above high school	165 (65.22)	92 (62.59)	
Occupation			0.251
White collar jobs	160 (63.24)	104 (70.75)	
Blue collar jobs	17 (6.72)	10 (6.80)	
Retired/Unemployed	76 (30.04)	33 (22.45)	
Household Income			0.829
<3000	42 (16.60)	22 (14.97)	
≥3000–4900	46 (18.18)	31 (21.09)	
≥5000–8999	57 (22.53)	33 (22.45)	
≥9000–14,999	56 (22.13)	27 (18.37)	
≥15,000	52 (20.55)	34 (23.13)	
Where do you live in Riyadh?			0.398
East	55 (21.74)	27 (18.37)	
West	53 (20.95)	22 (14.97)	
North	56 (22.13)	39 (26.53)	
South	58 (22.92)	33 (22.45)	
Middle	25 (9.88)	22 (14.97)	
Rural	6 (2.37)	4 (2.72)	
Type of Oral Health Problem			
Toothache	203 (80.24)	103 (70.07)	0.020 *
Gingival Bleeding	94 (37.15)	44 (29.93)	0.142
Abscess (pus)	55 (21.74)	13 (8.84)	0.001 *
Mouth Ulcer	24 (9.49)	13 (8.84)	0.830
Gingival Swelling	30 (11.86)	26 (17.69)	0.105
Halitosis (bad breath)	26 (10.28)	16 (10.88)	0.848
Other	7 (2.77)	18 (12.24)	0.0002 *

* *p*-value < 0.05 considered significant.

**Table 2:** Predictors for practicing self-medication.

Risk Factors of Self-Medication	OR	95%CI	*p*-Value
Gender (Males vs. Females)	0.691	0.428–1.116	0.130
Age	0.988	0.964–1.013	0.340
Nationality (Saudi vs. non-Saudi)	1.445	0.814–2.564	0.208
Marital Status (Married vs. Single)	1.071	0.642–1.784	0.793
Education (Above high school vs. High school or below)	1.309	0.766–2.236	0.324
Occupation (Blue collar vs. Retired/unemployed)	1.031	0.381–2.786	0.704
Occupation (White Collar vs. Retired/unemployed)	0.745	0.437–1.270	0.280
Income (≥5000–8999 vs. <3000)	0.675	0.316–1.443	0.769
Income (≥15,000 vs. <3000)	0.500	0.215–1.161	0.136
Income (≥3000–4900 vs. <3000)	0.630	0.300–1.326	0.574
Income (≥9000–14,999 vs. <3000)	0.907	0.401–2.050	0.330
Gingival bleeding (yes vs. no)	1.873	1.116–3.146	0.017 *
Toothache (yes vs. no)	2.008	1.172–3.438	0.011 *
Abscesses (yes vs. no)	3.243	1.593–6.602	0.001 *
Mouth ulcer (yes vs. no)	1.231	0.535–2.835	0.624
Gingival swelling (yes vs. no)	0.453	0.231–0.890	0.021 *
Bad breath (yes vs. no)	0.769	0.349–1.697	0.516

* *p*-value < 0.05 is considered significant.
